# Growth‐Like Swelling of 2D Precursors for Efficient Fabrication of Complex 3D Microstructures

**DOI:** 10.1002/advs.202520757

**Published:** 2026-03-05

**Authors:** Xinkai Zhu, Liang Wang, Xiangming Li, Chuanhang Zeng, Tianxiang Lan, Guifang Liu, Yangfan Qiu, Junjiang Li, Qi Chen, Bangbang Nie, Hongmiao Tian, Xiaoliang Chen, Chunhui Wang, Jinyou Shao

**Affiliations:** ^1^ Micro‐/Nano‐Technology Research Center State Key Laboratory for Manufacturing System Engineering Xi'an Jiaotong University Xi'an Shaanxi China; ^2^ Frontier Institute of Science and Technology (FIST) Xi'an Jiaotong University Xi'an Shaanxi China; ^3^ National Local Joint Engineering Research Center for Precision Surgery and Regenerative Medicine The First Affiliated Hospital of Xi'an Jiaotong University Xi'an Shaanxi China; ^4^ Interdisciplinary Research Center of Frontier Science and Technology Xi'an Jiaotong University Xi'an Shaanxi China; ^5^ School of Mechanics and Safety Engineering Zhengzhou University Zhengzhou China

**Keywords:** 3D microstructures, swelling, underwater adhesion, UV Cross‐linking

## Abstract

3D microstructures have offered the potential for performance enhancement in nearly every type of microsystem. Although traditional solvent‐induced swelling can easily and efficiently fabricate some complex 3D microstructures, their 3D morphology can be maintained only in a solvent environment, which severely limits the applications of these 3D microstructures. Here, we present a fabrication method to induce photocurable monomers into elastomeric microstructures to generate a buckling deformation of the microstructures, which is stable and irreversible after photo‐curing. This process is applicable to a variety of elastomeric polymers, photocurable monomers, and solvents. A 4‐inch sample of these 3D microstructures can be efficiently fabricated within 5–20 min. Moreover, it is compatible with imprinting processes, thereby further expanding both the material applicability and manufacturing efficiency for 3D microstructures. Meanwhile, a variety of complex 3D microstructures are obtained by controlling the growth‐like swelling cycles and the shape of 2D precursors. To demonstrate the enormous potential of this growth‐like swelling process, we use this strategy to fabricate octopus‐inspired 3D micro‐sucker arrays for underwater adhesion.

## Graphical abstract

The growth‐like swelling process fills photocured polymers into the elastomeric microstructures, inducing a significant swelling deformation of microstructures that can be maintained in the absence of solvents. The growth‐like swelling process breaks through the dependence of swelling deformation on the solvents, thereby expanding the application of 3D microstructures fabricated using swelling deformation.

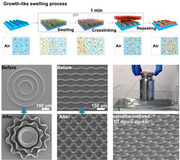

## 1 Introduction

Complex 3D microstructures in advanced functional materials are attracting increasing interest, as they allow for greater diversity in the design of desired structures and bring substantially enhanced performances, higher integration, and novel functionalities to the system. Therefore, complex 3D microstructures have been envisioned in nearly every type of micro/nano‐system technology, including flexible electronics [1, 2, 3, 4, 5], metamaterials [6, 7, 8, 9], micro‐electro‐mechanical systems [10, 11, 12], and microfluidic devices [13, 14, 15, 16]. A number of techniques have been developed for the fabrication of 3D microstructures, which can be broadly classified into two types: direct fabrications and indirect fabrications. Direct fabrications include complex modern micro‐machining technologies (such as the Bosch process) and various 3D printing technologies [17, 18, 19, 20, 21]. However, these technologies generally have the problems of high cost and low efficiency, especially as the complexity of the structure increases, the required cost and time also increase exponentially. Furthermore, there is a trade‐off between printing resolution and printing efficiency: higher resolution typically results in a smaller printable area and slower printing speed [21, 22, 23].

In contrast, the mature planar manufacturing technologies offer low cost, high output, and large‐area production while maintaining high precision [24, 25]. Therefore, utilizing planar processes to produce 2D precursors and assembling them (e.g., via rolling [26, 27], folding [28, 29], bending [30, 31], and buckling [7, 32, 33, 34, 35]) to indirectly fabricate complex 3D microstructures has become a strategic research direction. Compared to other methods, the buckling‐guided methods enable broader 3D geometries as they integrate carefully designed 2D precursor structures, achieving more complex deformations during assembly [24].

The buckling‐induced deformation method relies on the accumulation of compressive internal stress; buckling deformation occurs when this compressive internal stress exceeds a critical buckling threshold. Among all stress loading methods, accumulating compressive internal stress via swelling‐induced volumetric expansion of constrained 2D precursors represents the simplest and lowest‐cost approach. Moreover, the compressive internal stress produced by swelling is typically isotropic, which is difficult to achieve through mechanical loading. When the elastomeric polymers with cross‐linked molecular chains come into contact with a solvent, their relaxed molecular chains between junction points are compelled to stretch, leading to an increase in volume [36, 37, 38]. When the elastomeric polymers are constrained, such a volume expansion generates a compressive internal stress and induces a buckling deformation, ultimately leading to the formation of unique 3D microstructures without the necessity to use expensive equipment or intricate operations [7, 9, 32, 33, 34, 35]. However, in previous research, swelling‐induced deformation of microstructures was sustained often within the solvent. Once the solvent is removed, the structural deformation vanishes, which severely limits the practical applications of such 3D microstructures. Therefore, a method that can inherit the simplicity and inexpensiveness of solvent‐induced buckling while simultaneously being capable of maintaining a stable 3D morphology regardless of the presence of the solvent would provide a remarkable strategy for fabricating 3D microstructures.

The combination of swelling and cross‐linking reactions appears to be a promising strategy. However, current research in this area has primarily focused on using photocurable monomers to swell flat polymer surfaces for generating surface wrinkles [39, 40, 41]. Nevertheless, the swelling capacity of photocurable monomers for elastic polymers (e.g., polydimethylsiloxane (PDMS)) is quite limited. Although the use of organic solvents to assist monomers infiltration into the elastomer network has been extensively studied in polymer blending for material modification [42, 43, 44, 45], this approach has not been sufficiently explored for fabricating complex and stable 3D microstructures with large deformations.

Here, we propose a strategy to achieve stable and irreversible swelling deformation by continuously introducing photocurable monomers into 2D precursors. Inspired by the biological growth also involves irreversible volumetric expansion through continuous material accumulation, we term this process “growth‐like swelling.” The growth‐like swelling deformation arises from the accumulation of photocured polymers in the microstructures, and the degree of swelling can be progressively increased by repeating the swelling cycle until saturation is reached. Notably, the photocurable monomers selectively swell the microstructures with negligible substrate swelling despite identical material composition, resulting in significantly enhanced buckling deformation of the microstructures. Furthermore, a 4‐inch‐diameter 2D precursor sample can be transformed into the target 3D microstructure within 5 to 20 min, without requiring any expensive equipment or materials. Moreover, this approach is suited to diverse elastomeric polymers, photocurable monomers, and solvents. Its compatibility with imprint patterning further expands the material choices and streamlines manufacturing. Finally, we demonstrate the potential of our strategy by fabricating octopus‐inspired 3D micro‐sucker arrays with excellent performance for underwater adhesion.

## 2 Results and Discussion

### 2.1 Mechanism of the Growth‐Like Swelling of the Microstructures

Although a variety of elastomeric polymers, photocurable monomers, and solvents can achieve growth‐like swelling, PDMS, ethyl acetate (EA), and polyethylene glycol diacrylate (PEG400DA) monomers were chosen as the primary materials to achieve higher efficiency and quality in fabricating 3D microstructures. PDMS is not only optically transparent and chemically stable but also exhibits nearly instantaneous swelling upon solvent contact, owing to its negligible long‐term polymer relaxation [45]. The initial PDMS 2D precursors are fabricated by the imprinting process (detailed in the Methods). Then, a mixed solution comprising organic solvents and photocurable monomers (including photoinitiators) is employed to induce growth‐like swelling of the microstructures. EA exhibits a strong swelling ability toward PDMS, with a swelling degree more than 10 times that of ethanol [45]. PEG400DA can readily dissolve in EA, forming a colorless and transparent mixed solution (Figure S1); furthermore, the polymer obtained from this mixed solution after ultraviolet (UV) curing remains colorless and transparent, which ensures a thorough reaction of the photocurable monomers within the microstructures.

**FIGURE 1:**
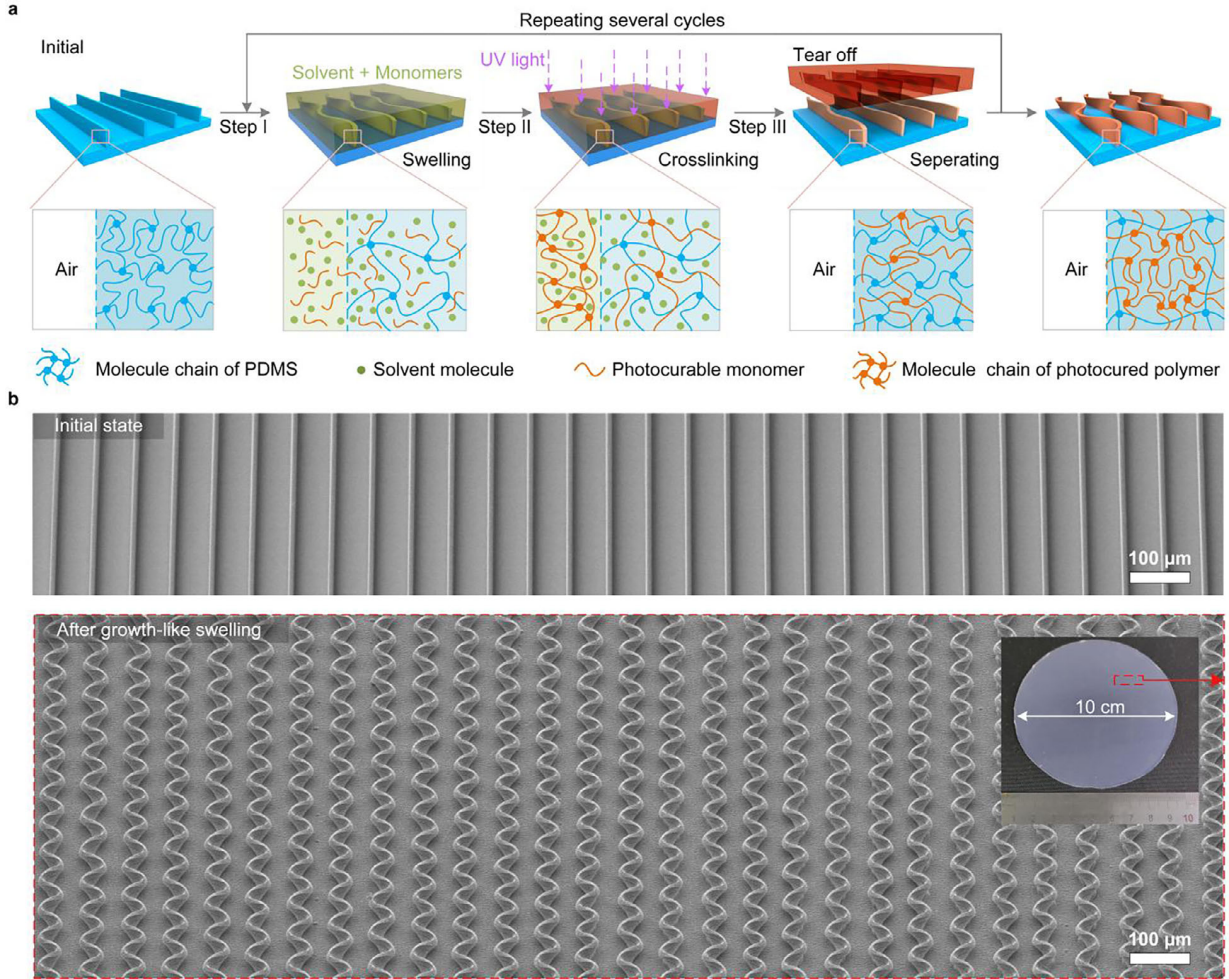
Mechanism of the growth‐like swelling. (a) Schematic of the growth‐like swelling process. (b) Side‐view scanning electron microscopy (SEM) images of the large‐area 3D microstructures after growth‐like swelling (bottom) and their initial microstructures (top); the inset is a photograph of the entire sample.

The mechanism of growth‐like swelling of PDMS microstructures is shown in Figure 1a. The cross‐linked molecular chains of the microstructures are forced to stretch due to the solvent‐induced swelling, increasing the opening diameter of the polymeric networks. As a result, the photocurable monomers have a high probability of diffusing into the microstructures (Step I). Subsequently, the photocurable monomers in the microstructures undergo cross‐linking reactions when subjected to UV irradiation (Step II). Although no chemical reaction occurs between the photocurable monomers and the PDMS, their molecular chains become intertwined, resulting in stable interpenetrating polymer networks. Therefore, after the solvent and the residual photocured polymers on the surface are removed (Step III), these photocured polymers remain in the microstructures. Such a growth‐like swelling cycle takes less than 1 min and does not require expensive equipment or complex operations.

The growth‐like swelling degree (i.e., the ratio of the volume change to the initial volume of the initial microstructures) can be enhanced by repeating the growth‐like swelling cycle. Due to the constraint imposed by the substrate, the growth‐like swelling can also lead to an increase in the compressive internal stress within the microstructures as the number of growth‐like swelling cycles increases. When such a stress exceeds a critical load, a buckling deformation occurs, and complex 3D curved microstructures are formed.

To validate the necessity of integrating photocurable monomers with organic solvents, we carried out a series of comparative experiments. EA rapidly causes a buckling deformation of the microstructures but evaporates quickly, which results in the complete disappearance of the deformation (Figure S2 and Movie S1). In contrast, no deformation is observed when using only the monomers (Figure S2c). Only the mixed solution can induce an irrecoverable deformation of the microstructures (Figure S2a).

### 2.2 Manufacturing Advantages of the Growth‐Like Swelling Process

Other elastomers, such as thermoplastic polyurethane (TPU) and Ecoflex silicone rubber, can also serve as 2D precursors for growth‐like swelling (Figure S3). Moreover, both TPU and Ecoflex silicone rubber can undergo buckling deformation when subjected to growth‐like swelling processes using different solvents, demonstrating the broad material applicability of our growth‐like swelling process (Figure S4).

Moreover, the 3D microstructures fabricated via the growth‐like swelling process exhibit excellent morphological stability. For instance, samples stored for over one year showed no significant change in their 3D morphology, demonstrating high temporal stability. Furthermore, the 3D morphology remained essentially unchanged after exposure to liquid‐nitrogen freezing or heating at 150 °C, indicating remarkable thermal stability. The microstructures also retained their shape even after being subjected to more than 2000 cycles of repeated mechanical stretching (Figure S5). Notably, even when severely damaged by strong acid or alkali corrosion, the remaining microstructures still preserved their 3D deformation (Figure S6).

The growth‐like swelling process enables the large‐area fabrication of 3D microstructures that combine multi‐scale feature sizes and high resolution. For instance, a 4‐inch 2D precursor sample can be transformed into 3D microstructures within 5 to 20 min (Figure 1b; Figure S7). This process does not require expensive equipment or materials. Furthermore, it can produce 3D microstructures with feature wavelengths spanning an impressive range from 14 µm to 2.2 mm (Figure S8). The capability for high‐resolution fabrication is further demonstrated by the 400 nm linewidth achieved in the 3D gratings shown in Figure S8a.

### 2.3 The Selectivity of Growth‐Like Swelling on the Microstructures

We observed that the photocured polymers were concentrated in the microstructure region but sparse in the substrate, likely due to differential swelling induced by the solvent on the substrate and the microstructures. According to mesh size theory, monomers can permeate only through sufficiently large openings in the PDMS network; swelling enlarges these openings, increasing permeation probability [36, 37, 38]. The microstructures, with their high surface‐to‐volume ratio and minimal constraint (anchored only at the base), undergo pronounced swelling, thereby facilitating high monomer permeation. In contrast, swelling of the PDMS substrate is limited. Its low surface‐to‐volume ratio and constraints from periodic boundaries restrict expansion primarily to the vertical direction. Moreover, the swelling of the microstructures compresses the surrounding substrate, further suppressing its in‐plane swelling. Consequently, the mesh openings within the substrate surface remain small horizontally, hindering monomers infiltration. Finite‐element analysis (FEA) confirm this mechanism, revealing high compressive internal stress at the substrate surface and significantly lower stress within the microstructures.

**FIGURE 2:**
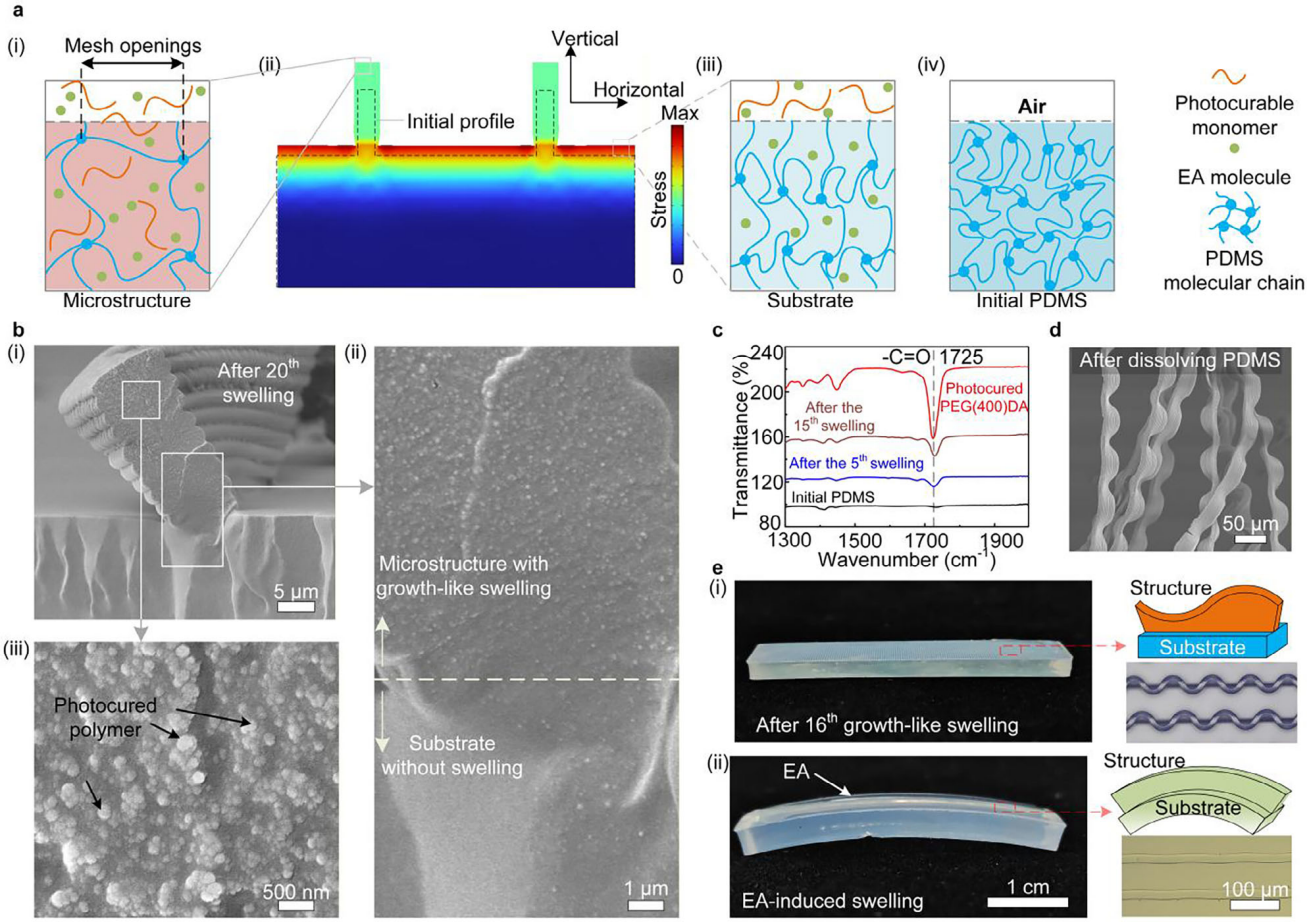
Photocurable monomers swelling the microstructures selectively. (a) Schematic of the photocurable monomers swelling the microstructures selectively and FEA showing the stress distribution in both the substrate and the microstructures after solvent‐induced swelling (ii). (b) SEM images of the microstructure cross‐section after the 20th growth‐like swelling cycle. (c) FTIR spectra of the PDMS sample as well as the photocured polymers and the PDMS sample after the5th and 15th growth‐like swelling cycles. (d) SEM image of the microstructures after dissolving PDMS. (e) Growth‐like swelling results in a more pronounced buckling deformation of the microstructures (i) compared with EA‐induced swelling (ii).

To analyze the distribution of photocured polymers, the cross‐section of the sample after growth‐like swelling, was examined (Figure 2b). Figure 2b(ii) shows a gradual transition in cross‐sectional morphology from the microstructure to the substrate region. The microstructure region exhibits a rough morphology with numerous dense phase‐separated particles embedded within it (Figure 2b(iii)), in contrast to the smoother substrate region. Furthermore, increasing the number of growth‐like swelling cycles results in a higher density of the phase‐separated particles within the microstructures (Figure S9). Time‐of‐flight secondary ion mass spectrometry (ToF‐SIMS) reveals a rising trend in the surface ester concentration with increasing numbers of swelling cycles (Figure S10).

To further confirm that these phase‐separated particles are indeed photocured polymers, we performed Fourier‐transform infrared (FTIR) spectra and energy‐dispersive X‐ray spectroscopy (EDS) of the samples. The FTIR spectrum of the samples treated with the growth‐like swelling process exhibited characteristic peaks unique to the PEG400DA, and the intensity of these characteristic peaks increased with the number of growth‐like swelling cycles (Figure 2c). Similar results were obtained using several different photocurable monomers (Figure S11). Additionally, the EDS results acquired from different regions of the sample reveal notably higher concentrations of C and O in the microstructures than in the substrate (Figure S12). Therefore, these data suggest that the phase‐separated particles may be a result of the thermodynamic incompatibility between the photocurable monomers and PDMS, which causes the self‐assembly of the photocurable monomers within PDMS [46, 47].

**FIGURE 3:**
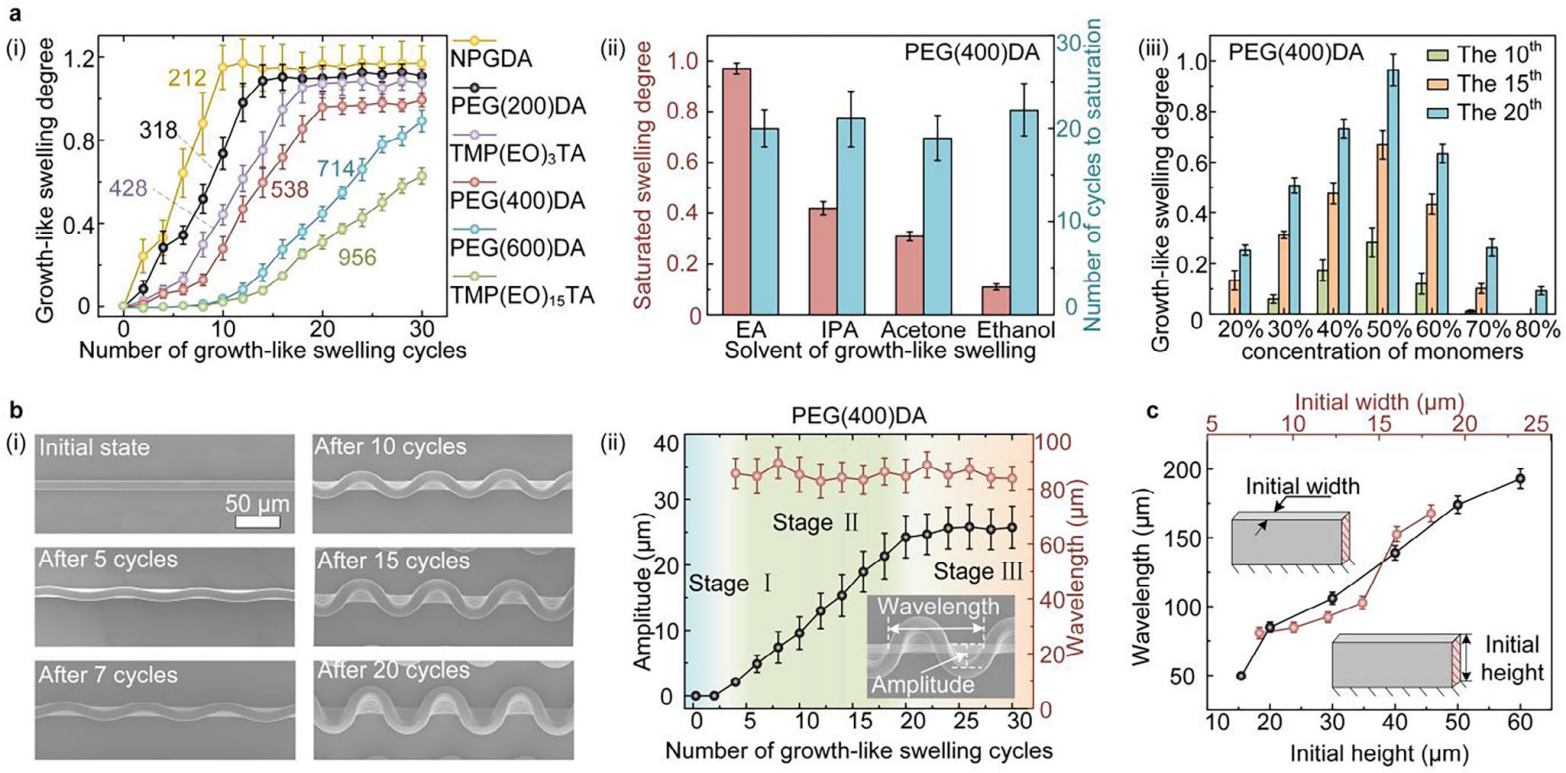
Control of the geometric parameters of the 3D microstructures. (a) Influence of the number of swelling cycles (i), molecular weight (i), and concentration (iii) of the photocurable monomers, and solvent (ii) on the degree of growth‐like swelling. The numbers in (i) correspond to the molecular weights of the photocurable monomers. (b) Amplitude and wavelength of the gratings after different numbers of growth‐like swelling cycles and corresponding SEM images. (c) Wavelength for different initial widths and heights. Data are presented as the mean ± s.d. (*n*  =  3 measurements from distinct samples in a, b, and c).

To more intuitively demonstrate that the photocurable monomers mainly penetrate into the microstructures, we treated the sample with a silicone rubber dissolving agent to remove the PDMS. After dissolution of the PDMS substrate, the microstructures remained intact with numerous micropores on their surfaces (Figure 3d; Figures S13 and S14).

The selectivity of the growth‐like swelling process for the microstructures induces more significant buckling deformation. In traditional EA‐induced swelling, the solvent also swells the substrate, thereby releasing the compressive internal stress in the microstructures (Figure 2e(ii)). Conversely, in the growth‐like swelling process, the compressive internal stress within the microstructures can only be relieved through buckling, leading to a pronounced deformation (Figure 2e(i)). Traditional EA‐induced swelling can generate a buckling deformation in gratings with an aspect ratio (the grating height‐to‐width ratio) greater than 3, while growth‐like swelling can generate a significant buckling deformation in gratings with an aspect ratio as small as 1.2 (Figure S15).

### 2.4 Control of the 3D Microstructure Geometric Parameters

Taking grating microstructures, the most typical microstructures, as an example, we systematically analyzed possible strategies for controlling the various geometric parameters of the 3D curved grating, including wavelength (i.e., the period), amplitude (i.e., the maximum displacement of the free‐end edge relative to the fixed‐end edge within a half‐wave unit).

We first investigated the effects of the number of growth‐like swelling cycles, photocurable monomers, and solvents on the growth‐like swelling degree. This degree increases with the number of swelling cycles and eventually reaches saturation. Moreover, when using monomers with smaller molecular weights, the growth‐like swelling degree of the PDMS microstructures increases more rapidly, and the saturation is slightly higher (Figure 3a(i)). To more rigorously analyze the content of monomers with different molecular weights within the microstructures, we performed FTIR on the surface of PDMS samples after 10 growth‐like swelling cycles and calculated their relative concentrations (Figure S16). Among them, NPGDA has the highest content, while TMP(EO)_15_TA has the lowest. This trend confirms that smaller molecules can more readily penetrate the cross‐linked network of the PDMS microstructures. The stronger the solvent's swelling capacity for PDMS in the mixed solution, the higher the saturated growth‐like swelling degree will be (Figure 3a(ii)). In addition, the concentration of photocurable monomers affects the growth rate of swelling degree, with the fastest increase observed at a proportion of 50% (Figure 3a(iii)).

The modulus of the PDMS 2D microstructures is also a critical factor for the growth‐like swelling deformation effect. By adjusting the ratio of the PDMS monomers to the cross‐linker, microstructures with different modulus were obtained (Figure S17a). We observed that PDMS microstructures with a lower modulus exhibited more pronounced buckling deformation (Figure S17b). However, those with an excessively low modulus were prone to structural damage due to their inferior mechanical properties. Overall, a monomers‐to‐crosslinker ratio of 10:1 achieved a balance, enabling rapid and significant deformation while incurring little structural damage.

The amplitude of the 3D curved gratings is significantly affected by the growth‐like swelling degree; thus, the amplitude can be effectively controlled by adjusting the number of swelling cycles (Figure 3b). Although the swelling in the first few swelling cycles (Stage I) also induces a compressive internal stress, this stress does not exceed the critical load due to the small aspect ratio of the grating (2.0). If the grating has a sufficiently large aspect ratio, a buckling deformation will occur after the first swelling cycle (for example, see the case of a grating with an aspect ratio of 5 in Figure S2a). In the amplitude‐growth stage (Stage II), the internal compressive stress within the microstructures exceeds the critical load, and the amplitude progressively increases with the number of swelling cycles. In the swelling‐saturation stage (Stage III), the amplitude no longer undergoes any significant change.

In contrast to the significant changes observed in the amplitude of the gratings, their wavelength remains nearly constant with increasing number of swelling cycles. This phenomenon may be attributed to the stress being concentrated at the center of the half‐wave unit when the obtained 3D curved gratings swell again (Figure S18). Although the wavelength of each individual half‐wave unit tends to increase with the number of swelling cycles, this trend is counteracted by the adjacent half‐wave units. However, the width and height of the initial gratings have a significant impact on the wavelength of the obtained 3D gratings (Figure 3c).

### 2.5 Compatibility with Imprinting Process

These 3D curved microstructures, fabricated through the growth‐like swelling process, are compatible with an imprinting process, enabling more efficient mass production and significantly broadening material compatibility. Although the physical properties of the PDMS microstructures, such as modulus (Figure S19) and surface reflectance (Figure S20), undergo significant changes after growth‐like swelling, the main objective of the growth‐like swelling process is to achieve unique 3D morphologies. Subsequently, through a two‐step imprinting process, these 3D microstructures can be replicated using materials better suited for specific applications.

**FIGURE 4:**
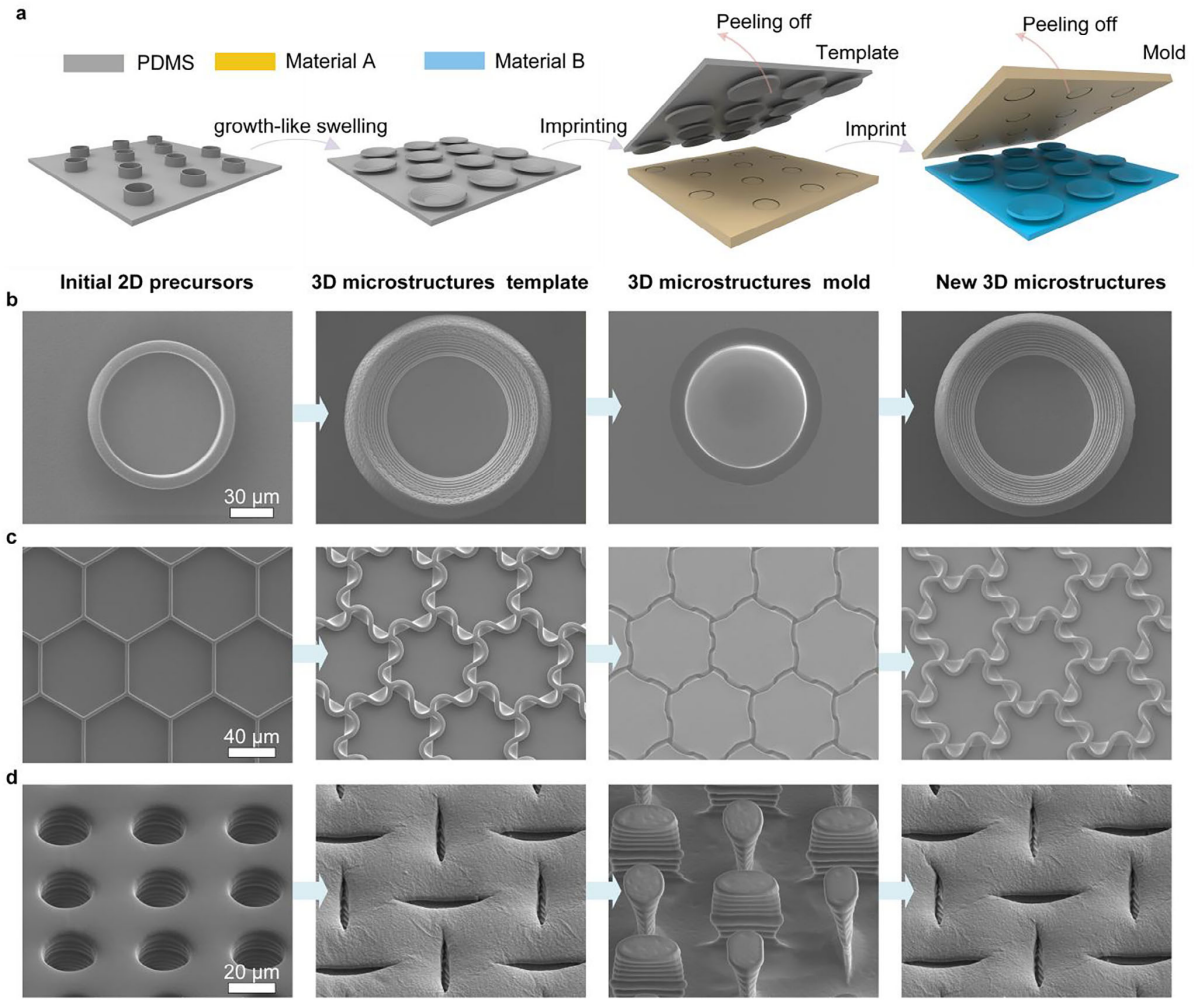
Compatibility of the growth‐like swelling process and imprinting process. (a) Schematic of the 3D microstructure replication process via the imprinting technique. Using the circular rings (b), regular hexagonal grids (c), and hole structures (d) as representative examples, the following are sequentially shown: SEM images of the 2D precursors, the resulting 3D microstructures after growth‐like swelling, the inverse structures obtained through imprinting, and the new 3D microstructure by a second imprinting step.

**FIGURE 5:**
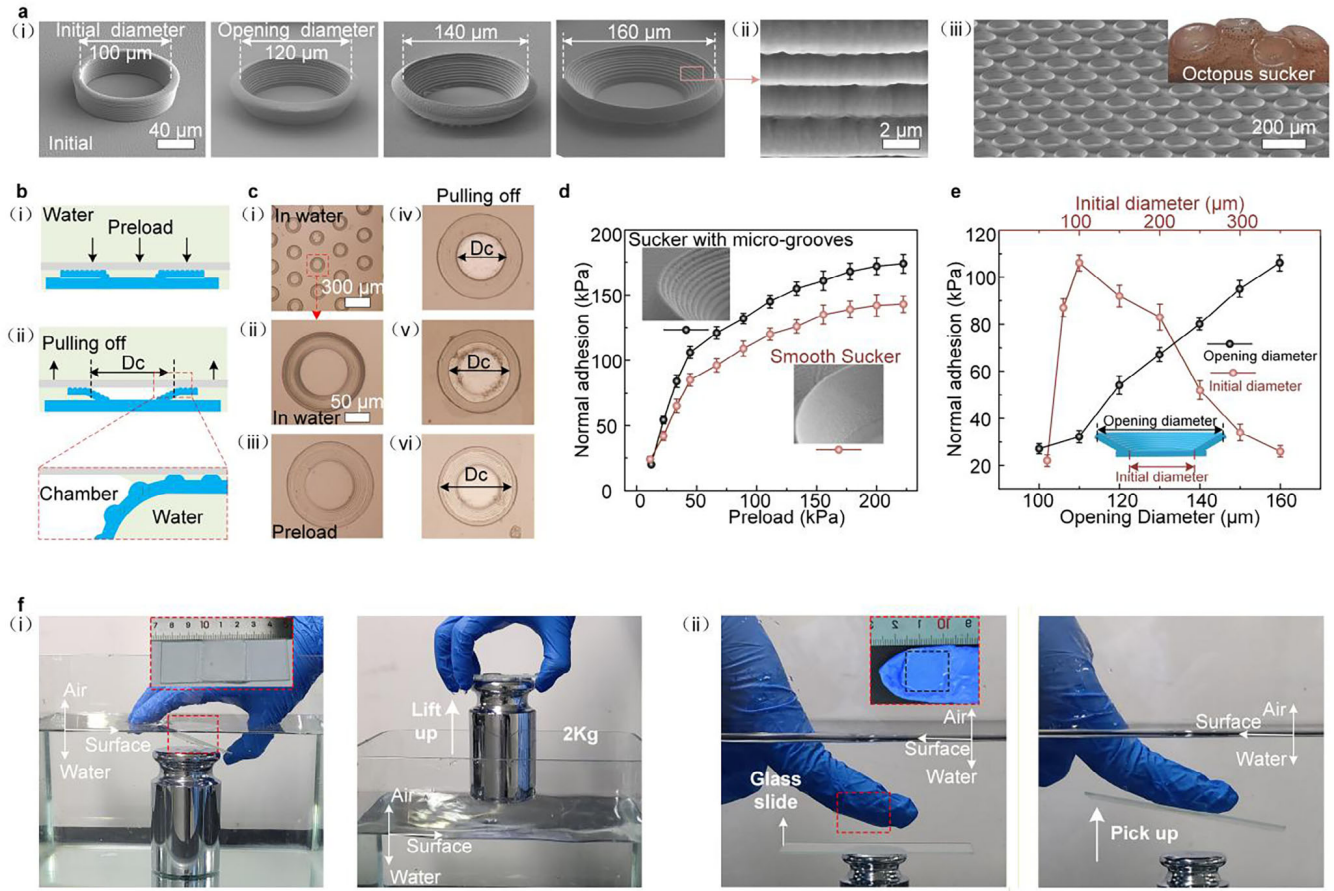
Octopus‐inspired 3D micro‐sucker arrays for underwater adhesion. (a) SEM images of the initial cylinder with a diameter of 100 µm and the 3D micro‐sucker with different opening diameters (i), microgrooves on the sidewall of the 3D micro‐sucker (ii), and 3D micro‐sucker array (iii). Inset: image of the octopus suckers. (b) Schematic of the adhesion mechanism of the 3D micro‐sucker. (c) Optical microscopy images of the adhesion and desorption processes. (d) Underwater adhesion of the 3D micro‐sucker arrays with and without microgrooves at different preloads (initial diameter = 100 µm, opening diameter = 160 µm, groove period = 2.2 µm). (e) Underwater adhesion of the 3D micro‐sucker arrays for different opening diameters and different initial diameters (preload = 40 kPa). (f) Photographs of the underwater adhesion process. A 2 kg weight can be lifted underwater and held in the air by a 25 × 25 mm^2^ 3D micro‐sucker array (i). A glass slide is picked up in water through a nitrile glove with a 15 × 15 mm^2^ 3D micro‐sucker array (ii). Data are presented as the mean ± s.d. (*n*  =  3 measurements from distinct samples in d and e).

The specific imprinting process for replicating 3D microstructures is shown in Figure 4a. First, a soft mold of the corresponding 3D microstructure is fabricated by imprinting with Material A (e.g., PDMS). Subsequently, material B is used to generate a new 3D microstructure based on this soft mold, and it can be chosen according to the intended application. For instance, in Figure 5b, the 3D micro‐sucker arrays are first imprinted using PDMS after hydrophobic treatment to create a 3D micro‐sucker mold. This mold is then used to fabricate the final underwater‐adhesive 3D micro‐sucker arrays through imprinting with a custom‐prepared imprinting resin. The resulting 3D microstructures exhibit excellent shape fidelity relative to the original 3D microstructures (Figure 4b). Additionally, we have also successfully fabricated a variety of complex 3D microstructures, including 3D curved microgrooves (Figure 4c) and 3D curved microcolumns (Figure 4d) through the imprinting process. To better demonstrate the material diversity enabled by the imprinting process for the 3D microstructures, we fabricated 3D micro‐sucker arrays using various materials, including PDMS, photocurable resin, TPU, and Ecoflex silicone rubber (Figure S21).

### 2.6 Octopus‐Inspired 3D Micro‐Sucker Arrays for Underwater Adhesion

Artificial adhesives can be attached to specific surfaces on demand and thus have significant potential in robotics, wearable electronic devices, and biomedical applications [48, 49, 50, 51, 52, 53, 54, 55, 56, 57, 58, 59, 60, 61, 62, 63]. Through swelling micro‐cylinder arrays, we fabricated an octopus‐inspired 3D micro‐sucker array template. Then, the 3D micro‐sucker arrays used for underwater adhesion were fabricated through the imprinting process (Figure 4a). The mechanical properties of the imprinting resin used to replicate the micro‐sucker array are presented in Figure S22. These 3D micro‐sucker arrays can also be fabricated on a large scale through this method (Figure 5a; Figure S23).

As the 3D micro‐sucker arrays are pulled from the adsorbed surface, a vacuum chamber is formed starting from the center of each micro‐sucker, which is analogous to the mechanism of the octopus suckers (Figure 5b). Additionally, the underwater adhesion performance of the 3D micro‐sucker arrays was improved by utilizing the microgrooves on the sidewalls (Figure 5a(ii)). These uniform annular microgrooves come from defects in the etching process of the silicon mold, while their geometry can be tuned by adjusting the etching parameters, and they exhibit high uniformity over large‐area samples (Figure S24).

These microgrooves discretize the sidewalls of micro‐suckers into multiple concentric sealing rings, which enable a gradual outward expansion of the vacuum chamber layer by layer of the sealing rings (Figure 5c), thereby achieving an effective sealing performance and a superior underwater adhesion compared to the smooth microsucker arrays. This result is consistent with several biological studies on octopus suckers and reports on the design of macroscopic suction cups [64, 65]. At a load of 30 kPa, the smooth sucker achieved an adhesion force of only 66 kPa. In contrast, the sucker with a groove period of 2.2 µm reached 84 kPa (Figure 5d), while the one with a groove period of 1.7 µm reached 91 kPa, representing an increase of 38% compared to the smooth sucker (Figures S25 and S26). The maximum underwater adhesion force of our 3D micro‐suckers was measured to be 172 kPa, which requires a preload of 200 kPa due to the material's high modulus. This performance remains competitive among reported octopus‐inspired 3D micro‐sucker arrays (Figure S27) [48, 49, 50, 51, 52, 53, 54, 55, 56, 57, 58, 59, 60, 61, 62].

Compared to the initial micro‐cylinders, the 3D micro‐suckers achieve a larger contact area with the adsorbed surface, resulting in better chamber sealing. In contrast, the initial micro‐cylinders not only have a limited contact area with the target surface but are also prone to structural deformation, which can lead to seal failure (Figure S28). As shown in Figure 5e, the adhesion force is only 29 kPa when the opening diameter is 100 µm (i.e., the initial micro‐cylinders) under a 40 kPa load. In comparison, a bowl‐shaped micro‐sucker array with a 160 µm opening achieves an adhesion force of 105 kPa—a 262% improvement—demonstrating the significant performance gain enabled by the 3D microstructures. Additionally, the initial diameters also significantly influence the adhesion performance of the 3D micro‐sucker arrays. The optimal underwater adhesion of these 3D micro‐sucker arrays occurs at 100 µm (the initial height is 25 µm, Figure 5e).

The superior underwater adhesion capability of the octopus‐inspired 3D micro‐sucker can be demonstrated through several experiments. A 25 × 25 mm^2^ 3D micro‐sucker array could attach to and lift a weight of 2 kg from water into the air (Figure 5f(i); Movie S2). Moreover, even after 600 cycles, the underwater adhesion force remained approximately 85% of the initial value (Figure S29), indicating excellent dynamic cycling stability. Furthermore, this 3D micro‐sucker array was capable of maintaining adhesion to heavy objects underwater over a week (Movie S3), exhibiting excellent static stability. Additionally, we imprinted a 15 × 15 mm^2^ micro‐sucker array onto a disposable nitrile glove, which could pick up a glass slide underwater (Figure 5f(ii); Movie S4). This lightweight and user‐friendly underwater pick method without any vacuum equipment offers greater convenience and flexibility than gripper‐based manipulation. It thus provides a potential solution for underwater operations.

## 3 Conclusions

In summary, we demonstrated that the photocurable monomers can easily diffuse into the microstructures when an organic solvent swells their polymeric network, generating stable 3D microstructures after UV curing that persist even after solvent removal. Interestingly, the selective diffusion of monomers into the microstructures induces more significant buckling deformation. Meanwhile, the growth‐like swelling degree can be controlled by adjusting the number of swelling cycles. Furthermore, the process is compatible with a variety of elastomeric polymers, photocurable monomers, and solvents that can achieve growth‐like swelling. The resulting 3D microstructures can be replicated in large quantities via imprinting, offering a highly efficient and scalable fabrication approach. Finally, we have successfully fabricated the 3D micro‐sucker arrays with exceptional underwater adhesion, which demonstrates the enormous potential of the growth‐like swelling process.

## 4 Experimental Section

### 4.1 Materials

The PDMS monomers and cross‐linkers (Sylgard 184) were obtained from Dow Chemical Co., Ltd. The photocurable monomers, including polyethylene glycol diacrylate (PEG200DA, PEG400DA, and PEG600DA), 1,4‐Butanediol dipropionate (NPGDA), ethoxylated trimethylolpropane triacrylate (TMP(EO)_3_TA and TMP(EO)_15_TA), 4‐hydroxybutyl acrylate (4‐HBA), prepolymers of epoxy acrylate (YC3390) and bisphenol A diglycidyl methacrylate (YC3380) were purchased from Shanghai Yinchang New Materials Co., Ltd. The photoinitiator 2‐hydroxy‐2‐methylpropiophenone (1173) and the prepolymer of amino acrylate (UN0003) were purchased from Shanghai Yinchang New Materials Co., Ltd. and Zhongshan Qianyou Chemical Materials Co., Ltd., respectively. EA (E809174, 99%), ethanol (E809061, 99.7%), and isopropyl alcohol (IPA) (I811932, ≥99.9%) were purchased from Shanghai Macklin Biochemical Technology Co., Ltd. Acetone (179124, ≥99.5%) was purchased from Merck KGaA, Darmstadt, Germany. The silicone rubber dissolving agent (GN‐6333, mainly composed of dichloromethane and benzenesulfonic acid) was purchased from Chongqing Guining Technology Co., Ltd.

### 4.2 Preparation of the PDMS Initial Microstructures

The PDMS initial microstructures were fabricated using a silicon template prepared by standard lithography and etching. The PDMS monomers were mixed with the cross‐linker in a mass ratio of 10:1. The resulting mixture was then coated on the silicon template. After vacuum degassing for a further 10 min, the PDMS was thermally cured at 120°C for 3 h in an oven. Finally, the PDMS sample was peeled off from the silicon template.

### 4.3 Preparation of the Mixed Solution

The photocurable monomers and photoinitiator were mixed at a 10:1 mass ratio and magnetically stirred for 10 h. The resulting mixture was then dissolved in EA and stirred for a further 20 min.

### 4.4 Fabrication of the 3D Microstructures through Growth‐Like Swelling

The mixed solution was injected onto the PDMS 2D precursors. It was then immediately exposed to UV light (300 W, 365 nm) for 30 s. Any residual photocured polymers on the surface of the PDMS sample were removed using tweezers. The desired 3D microstructures were obtained by repeating the above process several times.

### 4.5 Fabrication of the 3D Micro‐Sucker Arrays for Underwater Adhesion

First, a template of the 3D micro‐sucker arrays was fabricated through the aforementioned growth‐like swelling process and deposited with an antiadhesive layer of fluorocarbon (C4F8) via inductively coupled plasma chemical vapor deposition. Subsequently, a new PDMS 3D negative mold was replicated through the imprinting process. Next, the imprinting adhesive (mainly composed of 4‐HBA, TMP(EO)15TA, and photoinitiator 1173) was coated on the PDMS mold and UV curing (300 W, 365 nm) for 2 min. Finally, the new 3D microsucker arrays were peeled off from the PDMS mold (Figure 4a).

### 4.6 SEM and EDS Measurements

The SEM images and EDS spectra of all samples in this study were obtained using a field‐emission scanning electron microscope (SU‐8010, Hitachi Co., Ltd).

### 4.7 FTIR Spectroscopy

The FTIR spectra of all samples were acquired using a Fourier‐transform infrared spectrometer (Nicolet iS10). The reference sample was prepared and contained 4.24 g NPGDA, 0.42 g photoinitiator 1173, 7.4 g PDMS, and 0.74 g cross‐linker. Spectral data were processed using Omnic software (Thermo Scientific) to extract the integrated areas of the characteristic absorption bands. The ester bond concentration (C_(–C = O)_) was calculated using the following equation: $C_{\left(-C=O\right)}=\left[1-\frac{(A_{1725}/A_{784})\mathrm{Mono}\mathrm{mer}}{\left(A_{1725}/A_{784}\right)}\right]\times 100\%$

$$c_{\left(-C=O\right)}=\left[1-\frac{{\left(A_{1725}/A_{784}\right)}_{M\mathrm{onomer}}}{{\left(A_{1725}/A_{784}\right)}_{0}}\right]\times 100\%$$

where (A_1725_/A_784_)_0_ represents the ratio of the peak areas at 1725 cm^−1^ and 784 cm^−1^ for the reference sample, while (A_1725_/A_784_)_Monomer_ denotes the peak area ratio for PDMS microstructures after 10 swelling cycles with different monomers. The ester concentration was then divided by the number of ester groups per monomer molecule to obtain the monomer concentration (C_Monomer_).

### 4.8 Time‐of‐Flight Secondary Ion Mass Spectrometry (ToF‐SIMS)

All mass spectra were acquired on a Time‐of‐flight secondary ion mass spectrometer (IONTOF M6) using a Bi_3_^+^ primary ion beam at an energy of 30 keV.

### 4.9 Measurements of the Stress–Strain Curves and Elastic Modulus

The PDMS samples were cut into the dog‐bone shape depicted in Figure S30. The two wider ends of the sample were clamped onto the tensile fixture. The sample was then stretched at a constant rate until it reached an elongation of 25% of its original length (4 cm). The resulting force and displacement data were recorded and converted into stress and strain, thereby generating the stress–strain curve. The elastic modulus was determined as the slope of the initial linear region, which was obtained by performing a linear fit to the data using Origin software.

### 4.10 Measurements of the Growth‐Like Swelling Degree of the Microstructures

To facilitate the measurement of geometric parameters of microstructures after growth‐like swelling, the gratings with a width of 36 µm and an aspect ratio of 0.56 were selected as the research object. Due to the low aspect ratio, the gratings only experienced a volume expansion without any buckling deformation. The length of the grating can be considered constant, so the growth‐like swelling degree can be represented by the expansion of the cross‐sectional area. The cross‐section of the grating after growth‐like swelling was trapezoidal, and the top side length (a) and height (h) could be directly measured after growth‐like swelling using a laser scanning confocal microscope (Olympus, OLS4000). The surface width of the grooves imprinted from the PDMS sample represents the bottom side length (b) of the trapezoidal cross‐section of the grating. Thus, the cross‐sectional area (*S_n_*) of the grating after the *n* ^th^ swelling cycle can be calculated as:

$$S_{n}=\left(a+b\right)*h/2$$

Therefore, the growth‐like swelling degree (*S_Dn_*) of the grating after the *n* ^th^ swelling cycle can be calculated as:

$${S_{D}}_{n}=\left(S_{n}-S_{0}\right)/S_{0}$$

The measurements were performed on four randomly selected microstructure samples from the samples, and then the average values and error bars were plotted (Figure 3a).

### 4.11 Measurements of the Geometric Parameters of the Microstructures

The height of all microstructures was measured using a laser scanning confocal microscope (Olympus, OLS4000). The other geometric parameters were measured from the top‐view SEM images. All measurements were performed on four randomly selected samples from the samples, and then the average values and error bars were plotted (Figure 3b,c).

### 4.12 Normal Underwater Adhesion Measurements

The sample with the 3D micro‐sucker arrays was fixed at the bottom of a culture dish filled with deionized water, and a 3 × 3‐mm^2^ silicon wafer was moved using a computer servo pull‐pressure test machine (PT‐1176, Baoda, China) to measure the normal underwater adhesion force of the sample. The preload was applied by controlling the displacement of the silicon wafer at a speed of 5 mm/min. After holding the preload for 5 s, the silicon wafer was pulled up at a speed of 5 mm/min until it was completely detached from the sample. All measurements were performed on four randomly selected samples, and then the average values and error bars were plotted (Figure 5d,e).

### 4.13 Finite‐Element Analysis (FEA)

To analyze the stress distribution of the substrate (Figure 2a(ii)) or the microstructures (Figure 4a) during swelling, the commercial COMSOL 5.4 software package was adopted for the FEA. Multi‐physical field coupling of thin material transfer and solid mechanics was used for the FEA. In the FEA, the elastic modulus of PDMS was set to 750 kPa, and its Poisson's ratio was set to 0.49. Periodic boundary conditions at both sides and spring constraints at the bottom were applied to the PDMS structures.

### 4.14 Statistical Analysis

All data were collected from at least three independent replicates. Mean values were calculated and were presented with standard deviations as error bars in the figures. Detailed descriptions are provided in the respective Figure captions.

## Author Contributions

X.Z., L.W., X.L., and J.S. conceived the idea. L.W., X.L., and J.S. supervised the project and provided the technical advice. X.Z., L.W., and X.L. designed the fabrication method, and X.Z. and C.Z. designed and conducted most of the growth‐like swelling experiments. T.L., Y.Q., and J.L. designed and conducted the imprinting process of the 3D microstructures. T.L., Q.C., and B.N. performed the numerical simulation. X.Z., L.W., X.L., and J.S. analyzed and interpreted the data. X.L. and J.S. supervised and directed the research. X.Z., L.W., X.L., H.T., X.C., and C.W. wrote the paper.

## Conflicts of Interest

The authors declare no conflicts of interest.

## Supporting information




**Supporting File 1**: advs74605‐sup‐0001‐SuppMat.docx.

**Supporting File 2**: advs74605‐sup‐0002‐Movie S1.mp4.

**Supporting File 3**: advs74605‐sup‐0003‐Movie S2.mp4.

**Supporting File 4**: advs74605‐sup‐0004‐Movie S3.mp4.

**Supporting File 5**: advs74605‐sup‐0005‐Movie S4.mp4.

## Data Availability

The data that support the findings of this study are available from the corresponding author upon reasonable request.
