# Supplementary material for: Growth‐Like Swelling of 2D Precursors for Efficient Fabrication of Complex 3D Microstructures
Source: Adv Sci (Weinh). 2026 Mar 5;13(27):e20757. doi: 10.1002/advs.202520757 (PMC13170244; doi:10.1002/advs.202520757)
Supplement: Supplementary file 1 — Supporting File 1: advs74605‐sup‐0001‐SuppMat.docx. [file ADVS-13-e20757-s004.docx]

Supporting Information

**Growth‑like Swelling of 2D Precursors for Efficient Fabrication of Complex 3D Microstructures**

*Xinkai Zhu, Liang Wang^*^, Xiangming Li^*^, Chuanhang Zeng, Tianxiang Lan, Guifang Liu, Yangfan Qiu, Junjiang Li, Qi Chen, Bangbang Nie, Hongmiao Tian, Xiaoliang Chen, Chunhui Wang, Jinyou Shao^*^*

E-mail: (L.W.) [liangwang@xjtu.edu.cn](mailto:liangwang@xjtu.edu.cn); (X.L.) xiangmingli@xjtu.edu.cn; (J.S.) jyshao@xjtu.edu.cn

X. Zhu, L. Wang, X. Li, C. Zeng, T. Lan, G. Liu, Y. Qiu, J. Li, Q. Chen, B. Nie, H. Tian, X. Chen, C. Wang, J. Shao

Micro-/Nano-Technology Research Center

State Key Laboratory for Manufacturing Systems Engineering

Xi’an Jiaotong University , Xi’an, Shaanxi 710049, China

L. Wang, X. Li, X. Chen, J. Shao

Frontier Institute of Science and Technology (FIST)

Xi’an Jiaotong University , Xi’an, Shaanxi 710049, China

X. Li, J. Shao

Interdisciplinary Research Center of Frontier science and technology

Xi'an Jiaotong University, Xi’an, Shaanxi 710049, China

L. Wang

National Local Joint Engineering Research Center for Precision Surgery and Regenerative Medicine

The First Affiliated Hospital of Xi’an Jiaotong University, Xi’an, Shaanxi 710049, China.

B. Nie

School of Mechanics and Safety Engineering

Zhengzhou University, Zhengzhou 450001, China

**Figure S1. Photograph of colorless and transparent solvent, monomers, and mixed solution.** The monomers of polyethylene glycol diacrylate (PEG400DA) were used as the photocurable monomers, which can readily dissolve in ethyl acetate (EA), yielding a colorless and transparent mixed solution.

**Figure S2.** **Optical microscope images of PDMS gratings immersed in the mixed solution, pure EA, and pure monomers. a**) This mixed solution can induce an irrecoverable deformation of the microstructures. **b**) EA rapidly causes a buckling deformation of the microstructures but evaporates quickly, which results in complete disappearance of the deformation. **c**) No deformation is observed when using only the monomers.

**Figure S3. Growth-like swelling using other materials as 2D microstructures precursors.** **a**) Photographs and corresponding magnified optical micrographs of TPU microstructures before and after the **growth-like** swelling process. **b**) Photographs and corresponding magnified optical micrographs of Ecoflex silicone rubber microstructures before and after the **growth-like** swelling process. Both elastomeric polymers exhibited significant buckling deformation after **growth-like** swelling.

**Figure S4. Optical microscopy images of 2D precursors made of TPU and Ecoflex silicone rubber after 10 growth-like swelling cycles in different solvents.** Both (a) TPU and (b) Ecoflex silicone rubber 2D precursors can undergo growth-like swelling with ethyl acetate (EA), isopropanol (IPA), and ethanol to generate 3D buckling deformation. These results demonstrate the general applicability of the growth-like swelling process across diverse elastomeric materials and solvents.

**Figure S5. Morphological stability of the 3D microstructures.** **a**) SEM image taken shortly after 15 cycles of growth‑like swelling, which corresponds to the sample shown in Figure 3b(i) in the main text. **b**) SEM image of the above-mentioned sample after more than one year, showing essentially unchanged morphology. SEM images of the above-mentioned sample after heating at 150 °C (**c**) or immersion in liquid nitrogen (**d**) for 3 h, both revealing minimal morphological change. (**e, f**) The morphology remains largely unchanged even after more than 2000 cycles of mechanical stretching.

**Figure S6. Corrosion of 3D microstructures in strong acid and alkali solutions.** Shown are SEM images of the 3D microstructure after 15 cycles of growth‑like swelling (**a**) and of samples after 2 h immersion in 0.5 wt.% H₂SO₃ (**b**), 5 wt.% H₂SO₃ (**c**), 0.5 wt.% NaOH (**d**), and 5 wt.% NaOH (**e**). The 3D microstructures immersed in 5 wt.% H₂SO₃ and 0.5 wt.% NaOH were severely damaged, yet the residual structures still retained their 3D deformation. The microstructure treated with 5 wt.% NaOH was completely dissolved, leaving only the microstructure root, indicating that the photocured polymers predominantly accumulated in the microstructure regions with minimal distribution in the substrate.

**Figure S7. Scalable fabrication of 3D curved gratings through growth‑like swelling.** The SEM top view image of a 4-inch sample of 3D curved gratings obtained through growth‑like swelling demonstrates the capability of scalable fabrication of the growth‑like swelling. Inset: Photograph of a four-inch PDMS sample with the 3D curved gratings.

**Figure S8. Multiscale and high-resolution fabrication of 3D curved gratings.** The wavelength range of the 3D microstructures generated through the growth‑like swelling process spans from 14μm (**a**) to 2.2mm (**b**). Notably, the 3D gratings in Figure (**a**) achieves a linewidth of merely 400 nm, demonstrating the high-resolution fabrication of the growth‑like swelling process.

**Figure S9. SEM images of cross-sectional morphology of the 3D microstructures after different numbers of growth‑like swelling cycles.** The morphology of the microstructures appears rough, and many dense phase-separated particles measuring less than 300 nm in diameter are embedded in the microstructures. Furthermore, an increase in the number of growth‑like swelling cycles results in a higher density of the phase-separated particles concentrated in the microstructures.

**Figure S10. Time‑of‑flight secondary ion mass spectrometry (ToF‑SIMS) of ester group content on the sample surface after different numbers of swelling cycles**. The original PDMS microstructure contains no ester groups; these functional groups are exclusively present in the photocurable material used (PEG400DA). The mass spectra clearly show that the surface ester content increases gradually with the number of swelling cycles, which is consistent with the results obtained from FFT analysis.

**Figure S11. FTIR spectra of PDMS samples before and after growth‑like swelling with several different photocurable monomers.** The FTIR spectra of PDMS samples, PDMS samples after 15 cycles of growth‑like swelling and photocured polymers: **a**) Amino acrylate; **b**) Epoxy acrylate; **c**) 4-hydroxybutyl acrylate (4-HBA); **d**) Bisphenol A diglycidyl methacrylate (Bis-GMA). There are distinct new characteristic peaks in the spectra of the PDMS samples after growth‑like swelling. Furthermore, these characteristic peaks are most prominent in the spectra of the photocured polymers and are absent in pure PDMS, indicating the incorporation of the photocured polymers into the PDMS.

**Figure S12. EDS results acquired from different regions of the sample.** The photocured polymers we use contain many C and O atoms but no Si atoms. The comparison of the EDS results acquired from different regions of the sample reveals notably higher concentrations of C and O in the microstructures than in the substrate, which means that photocured polymers are more concentrated in the microstructure region and less concentrated in the substrate region.

**Figure S13. Substrate-free 3D microstructures after dissolving PDMS.** **a**)The PDMS sample after 15 cycles of growth‑like swelling is immersed in silicone rubber dissolving agent. Inset: SEM image of 3D curved gratings on the PDMS samples. **b**) After the PDMS substrate is completely dissolved, the remaining polymer silk is transferred to ethanol for cleaning. **c**) The polymer silk is dried in air. Inset: SEM image of the polymer silk. The microstructures remained intact after the substrate was completely dissolved in the silicone rubber dissolving agent, which confirmed that the photocurable monomers selectively swell the microstructures. In this way, we obtained a method for fabricating substrate-free 3D microstructures.

**Figure S14. SEM images of the 3D microstructures after dissolving PDMS.** The microstructure surface before PDMS dissolution is relatively smooth, while many micropores appear on the surface of the microstructures after dissolving PDMS.

**Figure S15. Comparison of buckling deformation between growth‑like swelling with EA and traditional solvent-induced swelling with EA.** Traditional solvent-induced swelling can generate a buckling deformation in gratings with an aspect ratio greater than 3, while growth‑like swelling can generate a significant buckling deformation in gratings with an aspect ratio as small as 1.2

**Figure S16. Comparison of monomer concentration in PDMS samples after 10 growth‑like swelling cycles.** The molar ratio of ester groups to PDMS repeating units is defined here as the ester concentration (C_-C=O_), while the molar ratio of the cured polymer's repeating units to PDMS repeating units is defined as the monomer concentration (C_Monomer_). Figure **a** shows a reference sample prepared from a blend of 0.02 mol NPGDA and PDMS containing 0.1 mol repeating units, corresponding to an ester concentration of 40%. Figures **b** to Figures **g** show the FTIR spectra measured after 10 growth‑like swelling cycles for photocurable monomers with five different molecular weights. The ester concentration was calculated based on the corresponding peak areas (see Methods for details).

**Figure S17. The influence of PDMS elastic modulus on the degree of growth‑like swelling. a**) Stress-strain curves of PDMS samples cured at 100°C for 1 hour, prepared using four different formulations of PDMS monomers and cross-linker. **b**) The degree of growth‑like swelling of the cured 2D microstructures for the different PDMS monomers and cross-linker formulations.

**Figure S18. Finite element simulation of re-swelling of microstructures that have already undergone deformation.** The maximum displacement occurs at the center of the half-wave unit. The stresses is concentrated at the center of the half-wave unit when the obtained 3D curved gratings swell again, which divide the 3D curved grating into periodic half-wave units. Although the wavelength of each individual half-wave unit tends to increase with the number of swelling cycles, this trend is counteracted by the adjacent half-wave units.

**Figure S19. The effect of growth‑like swelling on the elastic modulus of PDMS.** With an increasing number of growth‑like swelling cycles, the elastic modulus of PDMS rises due to the infiltration of the photocured polymers, an effect likely attributable to the formation of an interpenetrating polymer network (IPN) between the photocured polymer and PDMS, which stiffens the material.

**Figure S20. The influence of growth‑like swelling on the surface reflectance of PDMS structures.** With an increasing number of growth‑like swelling cycles, the surface reflectance of PDMS progressively rises. This is likely because the photocured polymer is thermodynamically incompatible with PDMS and aggregates into nanoparticles inside it. The enhanced Mie scattering resulting from these nanoparticles leads to the increase in reflectance.

**Figure S21. A 3D micro-sucker array sample fabricated via the imprinting process using various materials.** Compatible with the imprinting process, the 3D micro-sucker array can be adapted to a wider range of materials through two-step imprinting, including PDMS, photocurable resin, TPU, and Ecoflex, further demonstrating the broader material applicability of these 3D microstructures.

**Figure S22. Stress-strain curve of the photocurable resin used for imprinting 3D micro-sucker arrays for underwater adhesion.** To enable the 3D micro-sucker arrays to exhibit superior underwater adhesion, we selected a photocurable resin possessing a higher elastic modulus for imprinting the new 3D micro-sucker arrays. The elastic modulus of this resin, calculated via linear fitting, is 7.1 MPa.

**Figure S23. SEM image of large-area 3D micro-sucker arrays fabricated by combining growth‑like swelling with imprint techniques.** The initial inner diameter is 100μm, the thickness is 6μm, the height is 25μm, the initial spacing is 100μm, and the opening diameter is 160μm. These 3D micro-sucker arrays can also be fabricated on a large scale by combining growth‑like swelling process with imprint techniques.

**Figure S24. The micro-suckers and their microgrooves exhibit good uniformity over a large area.** The microgrooves are inherently formed during the fabrication of the 2D silicon mold, resulting from the cyclic alternation of etching and deposition steps in the deep reactive ion etching (DRIE) process. These grooves exhibit excellent uniformity in their geometric parameters across a large area.

**Figure S25. 3D micro-sucker with different groove periods.** In the Bosch process, the period of the micro-grooves is determined by the processing depth per cycle, while the spacing of micro-grooves is tuned by the ratio of etch time to deposition time. When the period is too small (a) or the spacing between micro-grooves is too narrow (b), the microgrooves and ridges appear non‑uniform. In contrast, when the micro-groove period is sufficiently large, the grooves exhibit good uniformity.

**Figure S26. Normal adhesion force of the 3D micro-suckers with different micro-groove periods under a 30 kPa preload.** The cup with a groove period of 0 represents the smooth micro-suckers. When the period is too small, a complete sealing ring may not form effectively, the adhesion force shows no improvement compared with that of a smooth sucker. In contrast, when the micro-groove period is sufficiently large, the adhesion force becomes markedly higher than that of the smooth sucker.

**Figure S27. Benchmarking of underwater adhesion performance against other reported octopus-inspired micro-suckers.** Comparison of the maximum underwater adhesion force (**a**) and the adhesion force under a 30 kPa preload (**b**) between this work and other reported studies [1-15].

**Figure S28. Structural failure of the micro-cylinder array under compression. a**) The initial micro-cylinder array. **b**) Under an applied load via a clean glass slide, the micro-cylinders undergo twisting and deformation, resulting in compromised chamber sealing.

**Figure S29. Excellent dynamic cycling stability of these 3D micro-sucker arrays.** Even after 600 cycles, the underwater adhesion force remained approximately 85% of the initial value Detailed in the Methods, which demonstrates the excellent dynamic cycling stability of this 3D micro-sucker arrays. Detailed testing process are provided in Experimental Section/Methods .

**Figure S30. Sample and tensile testing machine for measuring the stress-strain curve and elastic modulus of materials. a**) A dog-bone shape elastomer polymer is secured in the universal testing machine and then stretched upward. The sample thickness is 2mm. The resulting force-extension curve is recorded, which is used to derive the stress-strain relationship. **b**) The two wider ends of the sample were clamped onto the tensile fixture.

**References**

[1]	M. N*.* Khan, T. Huo, Q. Zhang, et al., “Synergetic adhesion in highly adaptable bio-inspired adhesive,” *Colloids and Surfaces B: Biointerfaces* 212 (2022): 112335, [https://doi.org/10.1016/j.colsurfb.2022.112335](https://doi.org/10.1016/j.colsurfb.2022.112335).

[2]	S*.* Baik, G. W. Hwang, S. Jeong, et al., “Bioinspired Microsphere-Embedded Adhesive Architectures for an Electrothermally Actuating Transport Device of Dry/Wet Pliable Surfaces” *ACS Applied Materials & Interfaces* 13 (2021): 6930-6940, https://pubs.acs.org/doi/10.1021/acsami.0c21847.

[3]	S. Baik, H. J. Lee, D. W. Kim, et al., “Capillarity-Enhanced Organ-Attachable Adhesive with Highly Drainable Wrinkled Octopus-Inspired Architectures,” *ACS Applied Materials & Interfaces* 11 (2019): 25674-25681, https://pubs.acs.org/doi/10.1021/acsami.9b05511.

[4]	H. Min, S. Jang, D. W. Kim, et al., “Highly Air/Water-Permeable Hierarchical Mesh Architectures for Stretchable Underwater Electronic Skin Patches,” *ACS Applied Materials & Interfaces* 12 (2020): 14425-14432, https://pubs.acs.org/doi/10.1021/acsami.9b23400.

[5]	D. W. Kim, H. Kim, G.-T. Hwang, et al., “Conformably Skin-Adherent Piezoelectric Patch with Bioinspired Hierarchically Arrayed Microsuckers Enables Physical Energy Amplification,” *ACS Energy Letters* 7 (2022): 1820-1827, https://pubs.acs.org/doi/10.1021/acsenergylett.2c00259.

[6]	S*.* Chun, D. W. Kim, S. Baik, et al., “Conductive and Stretchable Adhesive Electronics with Miniaturized Octopus-Like Suckers against Dry/Wet Skin for Biosignal Monitoring,” *Advanced Functional Materials* 28 (2018): 1805224, https://doi.org/10.1002/adfm.201805224.

[7]	D. W*.* Kim, S. Baik, H. Min, et al., “Highly Permeable Skin Patch with Conductive Hierarchical Architectures Inspired by Amphibians and Octopi for Omnidirectionally Enhanced Wet Adhesion,” *Advanced Functional Materials* 29 (2019): 1807614, https://doi.org/10.1002/adfm.201807614.

[8]	Y*.* Wu, X. Li, H. Tian, et al., “ Microtemplated Electrowetting for Fabrication of Shape-Controllable Microdomes in Extruded Microsucker Arrays toward Octopus-Inspired Dry/Wet Adhesion,” *Advanced Functional Materials* 33 (2022): 2210562, https://doi.org/10.1002/adfm.202210562.

[9]	C. B. Dayan, S. Chun, N. Krishna-Subbaiah, et al., “3D Printing of Elastomeric Bioinspired Complex Adhesive Microstructures,” *Advanced Materials* 33 (2021): 2103826, https://doi.org/10.1002/adma.202103826.

[10]	Y. W. Lee, S. Chun, D. Son, et al., “A Tissue Adhesion-Controllable and Biocompatible Small-Scale Hydrogel Adhesive Robot,” *Advanced Materials* 34 (2022): 2109325, https://doi.org/10.1002/adma.202109325.

[11]	S. Baik, J. Kim, H. J. Lee, et al., “Highly Adaptable and Biocompatible Octopus-Like Adhesive Patches with Meniscus-Controlled Unfoldable 3D Microtips for Underwater Surface and Hairy Skin,” *Advanced Science* 5 (2018): 1800100, https://doi.org/10.1002/advs.201800100.

[12]	R. Huang, X. Zhang, W. Li, et al., “Suction Cups-Inspired Adhesive Patch with Tailorable Patterns for Versatile Wound Healing,” *Advanced Science* 8 (2021): 2100201, https://doi.org/10.1002/advs.202100201.

[13]	G. W. Hwang, H. J. Lee, D. W. Kim, et al., “Soft Microdenticles on Artificial Octopus Sucker Enable Extraordinary Adaptability and Wet Adhesion on Diverse Nonflat Surfaces,” *Advanced Science* 9 (2022): 220202978, https://doi.org/10.1002/advs.202202978.

[14]	W*.* Li, X. Hu, H. Liu, et al., “3D light-curing printing to construct versatile octopus-bionic patches,” *Journal of Materials Chemistry B* 11 (2023): 5010-5020, https://pubs.rsc.org/en/content/articlelanding/2023/tb/d3tb00590a.

[15]	D, Lim, M. W. Jeong, H. Min, et al., “Autonomous self-healing 3D micro-suction adhesives for multi-layered amphibious soft skin electronics,” Infomat 6 (2024): e12603, https://doi.org/10.1002/inf2.12603.
